# Utilizing artificial intelligence for the diagnosis of ocular surface squamous neoplasia with ultrasound biomicroscopy images

**DOI:** 10.1007/s00417-025-07034-x

**Published:** 2026-01-10

**Authors:** Kubra Serbest Ceylanoglu, Zhao Zhenyang, Bernadete Ayres, Yike Li, Hakan Demirci

**Affiliations:** 1 Department of Ophthalmology, University of Health Sciences, Ankara City Hospital, Ankara, Türkiye Turkey; 2 Department of Ophthalmology, Kellogg Eye Center, University of Michigan, 1000 Wall Street, Ann Arbor, MI 48105 USA; 3 Emory Eye Center, Emory University, Atlanta, GA USA; 4 Department of Otolaryngology-Head and Neck Surgery, Vanderbilt University Medical Center, 1215 21 st Avenue South, Nashville, TN 37232 USA

**Keywords:** Ocular surface tumor, Conjunctival intraepithelial neoplasia, Conjunctival squamous cell carcinoma, Artificial intelligence, Convolution neural network

## Abstract

**Purpose:**

This study aims to develop an artificial intelligence (AI) model to assist ophthalmologists in distinguishing ocular surface squamous neoplasia (OSSN) from benign ocular surface lesions using ultrasound biomicroscopy (UBM) images.

**Methods:**

Data were retrospectively collected from 139 patients with biopsy-proven conjunctival lesions, including 201 UBM images of benign lesions (e.g.,pterygium, squamous papilloma) and 381 images of OSSN (e.g.,squamous cell carcinoma, conjunctival intraepithelial neoplasia). Patients with conjunctival pigmented lesions, melanoma, lymphoma, or those without a pathological diagnosis were excluded. UBM images were cropped to the anterior segment region and rescaled to a standard size of 300 × 200 pixels. Data augmentation techniques were applied to enhance the diversity of training images. A convolutional neural network was trained and tested using five-fold cross-validation. A heatmap was generated to illustrate the model’s decision-making process. The AI model’s performance was compared to that of three human experts with varying levels of experience. Additionally, univariate regression analysis was performed to assess the impact of patient-related factors (age, sex, race/ethnicity, lesion location, and side) on model performance.

**Results:**

Our AI model achieved an accuracy of 74.3 ± 3.9%, sensitivity of 75.0 ± 8.6%, specificity 73.0 ± 11.5%, precision of 83.3 ± 4.8%, F1 score (i.e., the harmonic mean of precision and recall) of 0.79 ± 0.06,and area under the receiver operating characteristic (AUROC) curve of 0.83 ± 0.03 in detection of OSSN. It significantly outperformed two ocular oncology fellows (*p* = 0.02 and 0.03, respectively) and demonstrated borderline significance compared to a senior ophthalmologist (*p* = 0.05). The heatmaps effectively highlighted the lesions, suggesting that echogenicity played a crucial role in the model’s predictions. None of the patient-related factors significantly affected model performance (all *p* > 0.1), supporting its equitable diagnostic capability across diverse patient groups.

**Conclusion:**

This study demonstrates the feasibility of using AI to differentiate OSSN from benign conjunctival lesions based on UBM images. The heatmap enhances model transparency, and the consistent performance across patient subgroups highlights its potential as a fair and valuable tool for clinical decision-making in ocular surface tumor evaluation.

## Key messages

*****What is known***:**

- Ocular surface squamous neoplasia (OSSN) and benign conjunctival lesions can have overlapping clinical features, making accurate diagnosis challenging. Ultrasound biomicroscopy (UBM) is a useful imaging tool, but its interpretation requires experience and expertise.

*****What is new***:**

- This study is the first to apply a convolutional neural network (CNN)-based artificial intelligence (AI) model to differentiate OSSN from benign ocular surface lesions using UBM images.
- The AI model demonstrated comparable or superior performance to human experts, especially outperforming ocular oncology fellows in diagnostic accuracy.
- Heatmap visualizations revealed that the AI model focused on lesion echogenicity, consistent with expert interpretation, suggesting improved transparency and clinical applicability.

## Introduction

Ocular surface squamous neoplasia (OSSN) is the most common malignancy of the ocular surface [1]. It is categorized into two main types: conjunctival intraepithelial neoplasia (CIN) and squamous cell carcinoma (SCC) [2]. The incidence of epithelial conjunctival tumors is rising, driven by increased exposure to ultraviolet light, higher human papillomavirus infection rates, and increased life expectancy [3]. Classic presentations of OSSN include elevated, papillary, gelatinous, or leukoplakic conjunctival lesions [3, 4]. However, clinical assessment alone is sometimes insufficient for diagnosis, as OSSN can closely resemble benign conditions such as pterygium, pinguecula, scar tissue and corneal pannus.

Distinguishing between benign and malignant ocular surface tumors is critical for timely treatment, better prognosis, and adequate follow-up. While histopathological examination remains the gold standard, non-invasive imaging techniques are increasingly used to facilitate earlier diagnosis and surgical planning. High-frequency ultrasound biomicroscopy (UBM) provides detailed visualization of the anterior segment, offering advantages such as visualization of the entire tumor, improved tumor margin assessment, reduced posterior tumor shadowing, enhanced image quality, and superior resolution for both pigmented and non-pigmented tumors [5, 6]. Despite its potential, UBM requires specialized training for accurate interpretation.

Convolutional neural networks (CNNs), a subset of deep learning, have gained significant attention in medicalimaging, particularly in ophthalmology. They process images through multiple layers: convolutional layers extract visual features such as edges, textures, and echogenic patterns; pooling layers reduce dimensionality while preserving essential information; and fully connected layers integrate these features to produce a final classification. These models can automatically learn graphical features and achieve high classification accuracy. Recent studies have successfully applied CNNs to detect anterior segment and retinal diseases using digital retinal photography and optical coherence tomography [7–9]. However, CNN-based analysis of ocular surface neoplasia has not yet been explored.

In this article, we aim to develop a deep learning model to differentiate OSSN from benign conjunctival lesionsusing UBM images. This technology could aid in the differential diagnosis of ocular surface lesions with similar clinical presentations, potentially improving early detection and clinical decision-making.

## Patients and methods

### Study population

This study adhered the tenets of the Declaration of Helsinki and received approval the University of Michigan’s ethics committee (#HUM00046408). A retrospective data review was conducted on patients who visited the Kellogg Eye Center between 2018 and 2023. Inclusion criteria required patients to (1) be 18 years or older, (2) have a confirmed pathological diagnosis of ocular surface lesions (including CIN, SCC, pterygium, pinguecula, actinic lesions, or squamous papilloma), (3) have available UBM images. The exclusion criteria included: (1) presence of other ocular surface lesions such as conjunctival melanoma, conjunctival lymphoma, or conjunctival pigmented lesions (including pigmented variants of OSSN), (2) history of prior ocular surface surgery or trauma, (3) previous treatment with topical or intralesional chemotherapeutic agents.

### Ultrasound biomicroscopy

UBM utilizes sound waves to analyze structures, similar to traditional ultrasound investigations. However, UBM employs a higher frequency, resulting in enhanced detail but limited tissue penetration [10]. All UBM examinations were performed by the same well-trained ophthalmologist (BA) using UBM 50 MHz equipment Absolu Quantel – Lumibird Medical (Cournon-d’Auvergne, France). The axial resolution was 30 μm, and the tissue penetration depth was approximately 5 mm [11, 12]. Subjects were examined in a supine position facing the ceiling in a dim lightroom. After topical anesthesia, a single-use water-filled bag with distilled water was used to separate the eyelids and produce a coupling medium between the transducer and the eye. The assessment included evaluating the epithelial characteristics of the lesion (thickness, echogenicity), stromal features (echogenicity, thickness, vascularity), and the regularity of the scleral border.

### Datapreprocessing

**Fig. 1:**
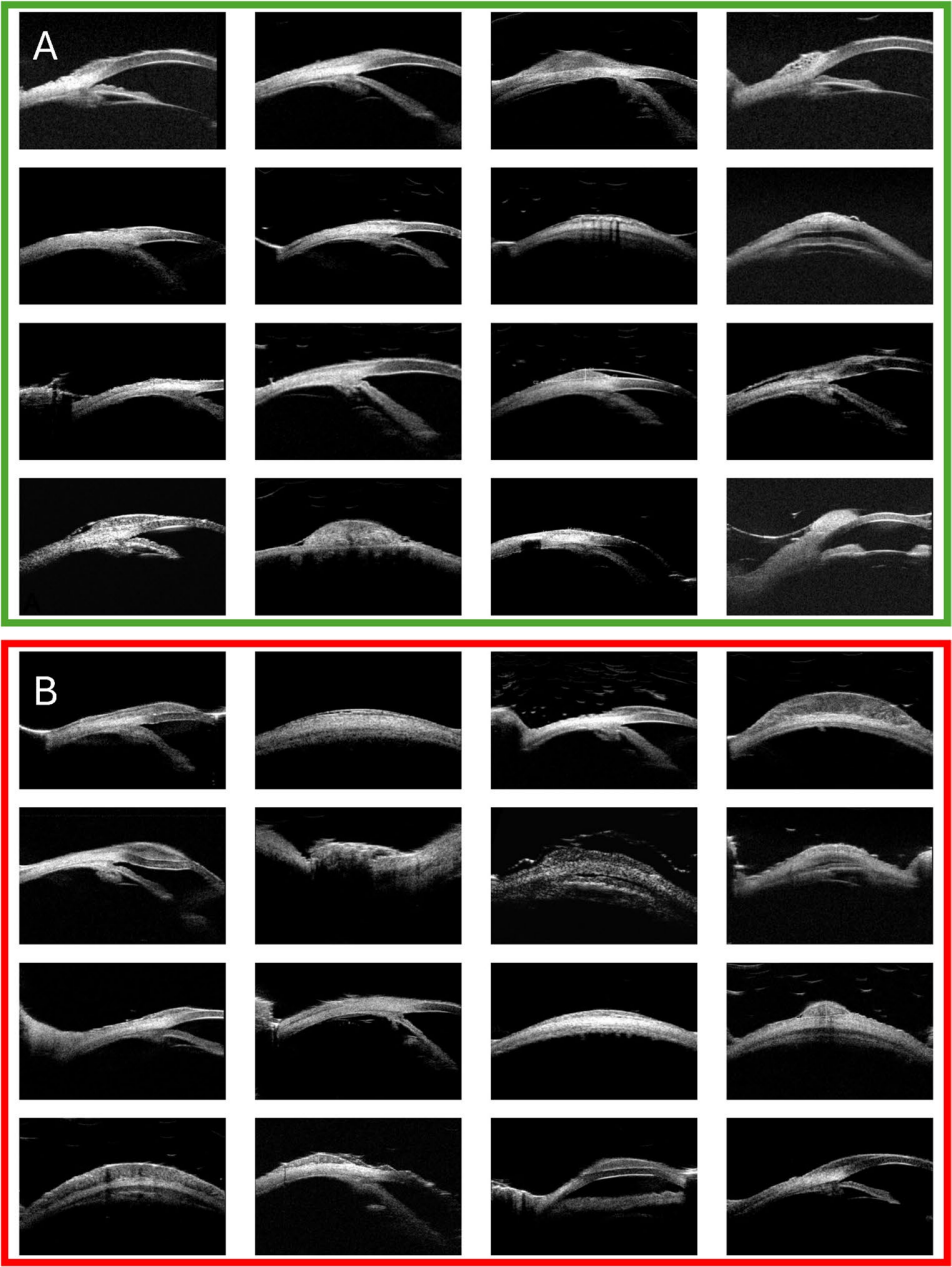
Examples of benign (**A**) and neoplastic (**B**) UBM images, as determined by pathology results

The UBM examination for each eye yielded approximately four images (range 3–8) from various angles or planes, resulting in a total of 582 images. These images were manually cropped to focus on conjunctival lesions and resized to dimensions of 300 × 200 pixels (Fig. 1). Data augmentation techniques, such as horizontal flipping and minor rotation, were used to expand and enrich the dataset. This process was randomly applied to each image during the model training phase.A ground truth label indicating whether the lesion was benign or malignant was assigned to each image based on the pathology report.

### Model development

The deep learning model employed in this research was constructed using Xception, an open-source CNN model developed by Google [13]. Xception represents an “extreme” version derived from the classical Inception model for image classification. Its distinctive architecture incorporates modified depth wise separable convolution layers with residual connections. This model functions by independently analyzing channel and spatial correlations in successive steps, thereby effectively reducing connection volumes and enhancing computational efficiency. Xception has exhibited remarkable performance, achieving 79.0% accuracy in top-1 and 94.5% accuracy in top-5 classifications on the ImageNet dataset, outperforming a majority of existing models. Additionally, the standard model boasts a relatively compact network size of 88 MB and operates with a brief average processing time of 8.1 milliseconds per inference step [14]. By virtue of these advantages, it has been increasingly applied in medical research, demonstratingpromising outcomes [15–19]. In this study, a simplified version of the Xception model was established, comprising 37 layers and 2.7 million parameters, with the top dense layer generating a two-class output.

The model was trained and tested in a binary classification task using a fivefold cross-validation approach. Specifically, the data were randomly split into an 80% training set (*n* = 465) and a 20% test set (*n* = 70). This process was iterated five times, producing five entirely distinct test sets. The data were separated in a stratified fashion to maintain a consistent distribution of classes in both the training and test sets. During training, a random selection of 20% data from the training set (*n* = 116) were employed for model validation.

Training was performed in Python v3.9 using TensorFlow v2.11. The learning rate was set at 0.0001 and regulated by the Adam optimizer [20]. Model performance was assessed by accuracy and categorical cross-entropy loss at each epoch. To prevent overfitting, the training session was terminated if no progress was observed in reducing validation loss over consecutive 15 epochs.

### Heatmap

**Fig. 2:**
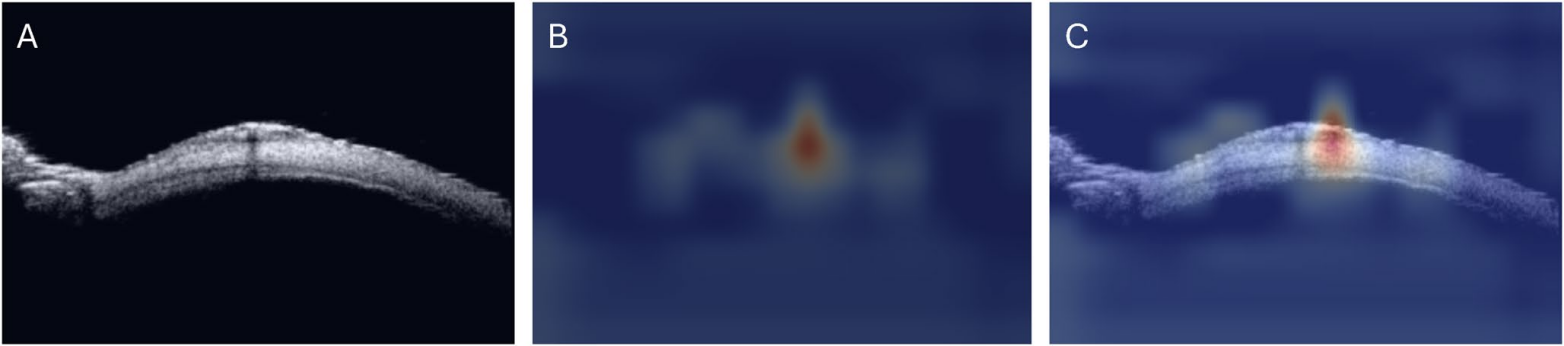
Example of gradient-weighted Class Activation Mapping (grad-CAM). UBM images (**A**) were visualized using grad-CAM (**B**), with highlighted areas indicating regions that the deep learning model considered important for identifying conjunctival lesions (**C**)

Gradient-weighted Class Activation Mapping (Grad-CAM) was utilized to visualize the model’s decision-making process (Fig. 2). In essence, this approach leverages the gradients of the target class within the final convolutional layer to weigh the importance of individual feature map channels. These weighted feature maps are subsequently combined using global average pooling, generating a coarse localization heatmap that accentuates crucial regions within the image [21]. Grad-CAM is widely recognized as an effective method for interpreting the decisions made by deep learning models in image classification tasks [22, 23]. In this study, heatmaps were generated to highlight findings associated with the predicted class and rescaled to match the original images.

### Benchmarking

No model has previously been reported to incorporate UBM images for assessing ocular surface lesions. To allow for an unbiased comparison, we recruited three human experts with varying levels of expertise to evaluate the samesubset of image data randomly selected from the pool (*n* = 139). The participants consisted of two ocular oncology fellows and one senior ophthalmologist specializing in ultrasound with 2, 2, and 15 years of experience, respectively. They were blinded to all cases including pathology. All images were de-identified, shufffled and presented to these experts for their independent diagnoses.

### Statistics

Descriptive statistics were applied as appropriate. The overall predictability of a model was evaluated by the area under the receiver operating characteristic (AUROC) curve. The optimal cut-off threshold on the curve was determined at the point with minimal distance to the upper left corner on the validation set and subsequently applied to the test set. The numbers of correctly and incorrectly classified cases were displayed in a confusion matrix, which was used to calculate the performance metrics, including accuracy, recall, specificity, precision and F1 score. The F1-score, which represents the harmonic mean of precision and recall, is particularly useful for evaluating performance in datasets with imbalanced classes. Results were averaged over 5 iterations of cross-validation and presented as mean ± standard deviation. Significance was determined through Cochran’s Q test between the AI model and human experts, with alpha set at 0.05. Additionally, univariate logistic regression was performed to evaluate whether demographic and clinical factors, such as age, sex, race/ethinicity, lesionlocation, and side were associated with model performance. Statistical analyses were conducted using Python 3.91.

## Results

The final dataset contained 139 patients (139 eyes) with a mean age of 68.5 ± 12.7 year, of which 81.1% were male. All patients had biopsy-proven conjunctival epithelial tumors, including 70 cases of CINs, 15 cases of conjunctival SCCs, and 54 benign tumors (Table 1).

**Table 1:** Overview of patient characteristics and lesion details

Characteristics	Patient count or mean (± SD)
Age (mean ± SD)	68.5 ± 12.7
Gender (F/M)	32/107
Side (right/left)	60/79
Ethnicity (n, %)	
White	130 (93.5%)
African American	4 (2.9%)
Hispanic	2 (1.4%)
Asian	2 (1.4%)
Indian	1 (0.7%)
Pathology	
Neoplastic conjunctival lesions	
CIN	70 (50.4%)
SCC	15 (10.8%)
Benign conjunctival lesions	
Pterygium	29 (20.9%)
Pinguecula	7 (5%)
Squamous papilloma	7 (5%)
Scar	3 (2.2%)
Actinic keratosis	7 (5%)
Epithelial hyperplasia	1 (0.7%)
Location	
Temporal	65
Nasal	46
Superior	9
Inferior	19

*F* female, *M* male, *CIN* conjunctival intraepithelial neoplasia, *SCC* squamous cell carcinoma, *SD* standard deviation

**Fig. 3:**
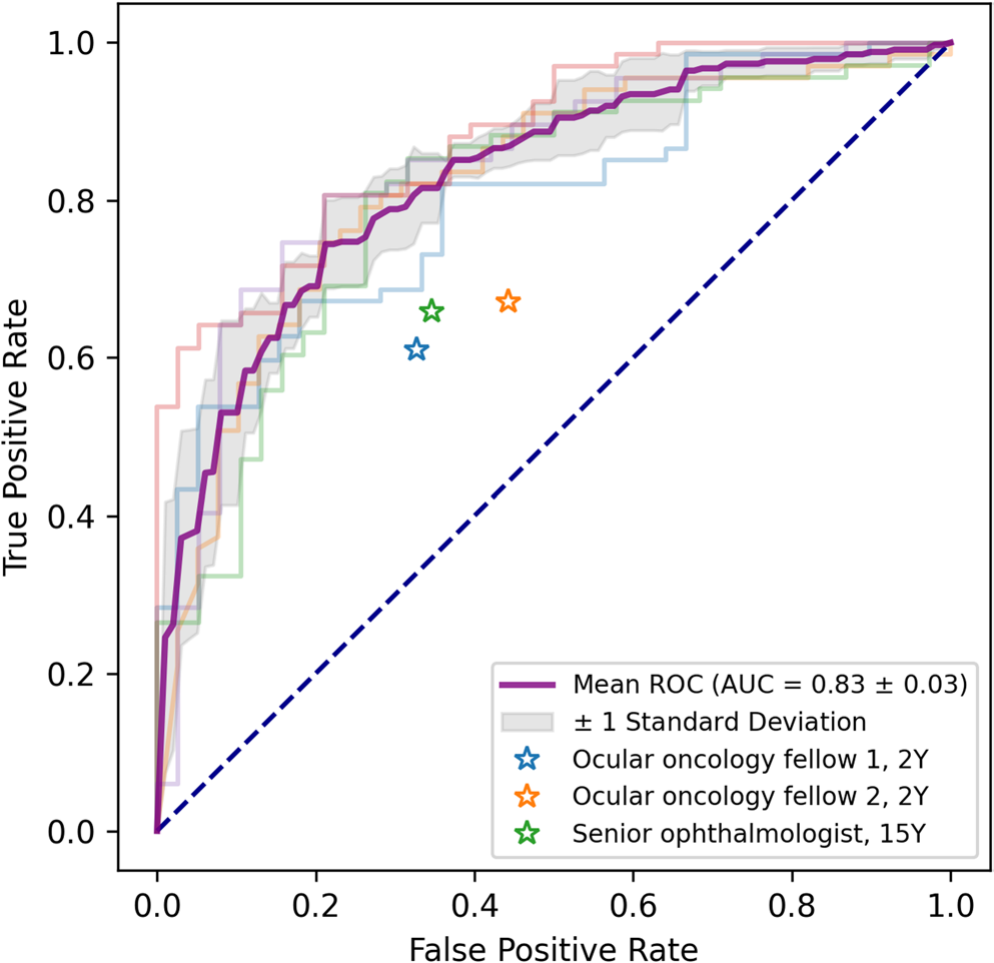
Receiver Operating Characteristic curves comparing the AI model and human specialists in differentiating between benign and neoplastic conjunctival tumors

The model achieved an accuracy of 74.3 ± 3.9%, a sensitivity of 75.0 ± 8.6%, a specificity of 73.0 ± 11.5%, a precision of 83.3 ± 4.8%, an F1 score of 0.79 ± 0.06, and an AUROC of 0.83 ± 0.03 in detecting CIN and SCC (Table 2; Fig. 3). Its performance was significantly better than that of the two ocular oncology fellows, who achieved accuracies of 63.4% (*p* = 0.02) and 62.7% (*p* = 0.03), respectively. The AI model also showed borderline significance compared to the senior specialist, who exhibited an accuracy of 65.7% and an F1 score of 0.70 (*p* = 0.05). None of the patient-related factors, including age, sex, race/ethnicity, lesion location, or side, significantly affected the model’s diagnostic performance (all *p* > 0.1).

**Table 2:** Comparison of classification performance between the proposed deep learning model and human experts

Method	Accuracy	Sensitivity	Specificity	Precision	F1 score	*p*
AI model	74.3 ± 3.9%	75.0 ± 8.6%	73.0 ± 11.5%	83.3 ± 4.8%	0.79 ± 0.06	
Rater 1 (2y)	63.43%	60.98%	67.31%	74.63%	0.67	0.02
Rater 2 (2y)	62.69%	67.07%	55.77%	70.51%	0.69	0.03
Rater 3 (15y)	65.67%	65.85%	65.38%	75.00%	0.7	0.05

**Fig. 4:**
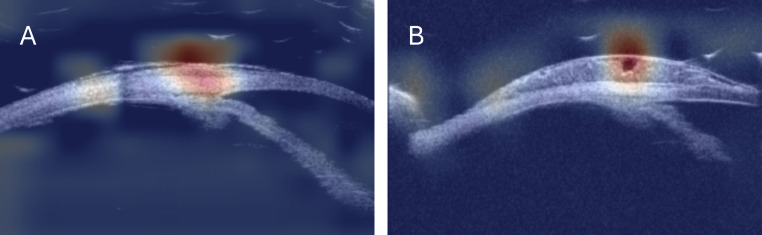
Examples of heatmaps in a benign case (**A**) and a neoplastic case (**B**). The AI model correctly diagnosed each case, with the heatmaps highlighting the critical regions that display uniform (**A**) and heterogeneous (**B**) echo patterns, which informed the decision

Heatmaps consistently highlighted the anterior segment regions with pathological findings characteristic of the lesions (Fig. 4). Visual assessment revealed that malignant lesions, such as OSSN, were associated with increased reflectivity and irregular internal architecture on UBM, while benign lesions exhibited more homogeneous and less reflective patterns. These observations suggest that echogenicity was a prominent feature utilized by the AI model during classification [24, 25].

## Discussion

This study highlights the potential of AI as a valuable tool for assisting ophthalmologists in the differential diagnosis of ocular surface tumors through a non-invasive approach. The AI model demonstrated decent performance in distinguishing benign ocular surface tumors from OSSN based on UBM images. It outperformed human experts in identifying clinically challenging lesions, which could lead to more accurate and timely diagnoses, improved patient outcomes, and better-informed surgical planning. Furthermore, the novel heatmap technique enhances the transparency of the AI’s decision-making process, offering deeper insight into its reasoning. These findings also underscore the value of UBM as an accessible and cost-effective imaging method for ocular surface lesion assessment. Moreover, the model’s diagnostic performance was not significantly influenced by patient- and tumor-related factors, suggesting equitable performance across diverse patient groups. This supports the potential of AI to reduce diagnostic disparities and promote fairness in clinical decision-making. They further support the role of AI technology in improving efficiency, reducing errors, and advancing precision medicine in healthcare in the era of big data.

While AI has been explored in ocular surface tumor detection, previous studies have primarily relied on anterior segment photographs without histopathological confirmation, potentially leading to diagnostic inaccuracies due to the clinical similarities between benign and malignant lesions [26–28]. Greenfield et al. utilized AI with high-resolution anterior segment optical coherence tomography (HR-OCT) images to differentiate OSSN from other conditions such as pterygium and pinguecula, achieving high accuracy (91.7%) and outperforming human graders [29]. This highlighted the potential of AI to enhance decision-making in OSSN diagnosis. However, their study included both clinically and biopsy-confirmed cases, introducing potential diagnostic uncertainty. In contrast, our study exclusively included biopsy-confirmed cases, establishing a more defined ground, reducing misclassification and improving model reliability. Additionally, while HR-OCT is shown to play an important role in OSSN detection, UBM offers deeper tissue penetration, making it a valuable tool for comprehensive tumor assessment. To our knowledge, this is the first study to apply deep learning to UBM images for conjunctival tumor classification using biopsy-confirmed data, making a significant step toward AI-driven precision diagnostics in ophthalmic oncology.

Previous studies have demonstrated the role of UBM in evaluating OSSN. Char et al. were the first to report the use of UBM for examining SCC of the ocular surface with intraocular extension [30]. On UBM, OSSN often appears hyperechoic on the surface, while hypoechoic internally, with characteristic findings such as anterior chamber angle blunting and uveal thickening, correlating strongly with histopathological observations. UBM was most helpful for evaluating and documenting the intraocular extension of OSSN [31]. Utilizing a non-invasive imaging modality like UBM to differentiate benign and neoplastic conjunctival lesions is crucial to avoiding unnecessary biopsies, achieving early and accurate diagnoses, guiding treatment strategies, and enhancing patient safety and comfort. A reliable preoperative prediction can significantly influence surgical management, including the choice of surgical technique, determination of excision margins, and the need for adjunctive therapies.

Our AI model’s average precision of 83.3% is particularly encouraging, as it suggests a low rate of false positives—an essential factor in diagnosing potentially life-threatening malignancies. The AUROC of 0.83 further supports the model’s ability to differentiate between benign and malignant lesions, indicating strong discriminatory power and highlighting AI’s potential as a valuable screening tool for ocular surface tumors. The clinical impact of our AI model can be multifaceted. Firstly, it can serve as a powerful diagnostic adjunct, aiding ophthalmologists in distinguishing between benign and neoplastic conjunctival lesions with greater confidence. Secondly, it may function as a reliable second opinion, particularly for general ophthalmologists or less experienced ocular oncologists, thereby reducing the possibility of misdiagnosis. Thirdly, in resource-limited settings, where access to ophthalmic oncologists or histopathological confirmation is limited, an AI-based system could provide preliminary diagnostic guidance, facilitating clinical decision-making and referrals. Fourthly, unlike human assessments, which can be influenced by fatigue, subjective interpretation, and variability in experience, a well-trained AI model offers standardized and reproducible evaluations. Finally, a dependable prediction of neoplasia risk could aid in personalizing treatment plans, determining excision margins, assessing the need for adjunctive therapies, and establishing appropriate follow-up protocols.

The Grad-CAM heatmaps provided valuable insights into our AI model’s decision-making process, highlighting the role of echogenicity in lesion identification. This approach aligns with expert interpretation of UBM images and reflects established diagnostic principles in ocular oncology [24, 25]. Malignant lesions such as OSSN often exhibit rapid, disorganized cellular proliferation, increased density, and histopathological features like necrosis and apoptosis. These factors can contribute to irregular reflectivity and internal architecture on imaging. In contrast, benign lesions typically display uniform tissue composition and cellular morphology, resulting in more homogeneous echogenic patterns [31, 32]. The AI model’s focus on these echogenic variations suggests it has learned to identify clinically meaningful features. The transparency offered by heatmaps enhances trust in the model’s predictions and facilitates collaboration between AI and clinicians. Moreover, heatmaps may serve as educational tools, helping less experienced practitioners refine their interpretation skills, which is particularly valuable in training settings or resource-limited environments. This study reinforces the importance of interpretable AI in bridging the gap between technological innovation and clinical expertise [22].

Despite these promising findings, several limitations must be acknowledged. First, this retrospective study was conducted on a relatively small, single-center cohort, which may have racial and geographical homogeneity among patients. It also focused on patients who underwent surgery for conjunctival tumors. To ensure broader applicability, further validation with more diverse, multi-center datasets is needed. Prospective studies will also be essential to evaluate the AI model’s real-world clinical utility. Second, our dataset exhibited a modest imbalance, with fewer benign lesions compared to neoplastic lesions, which could influence model performance. Expanding the dataset to include a wider range of benign lesions would enhance model robustness. Third, only a single deep learning model was tested, without an ablation study to optimize network architecture. Future research should explore alternative model structures and conduct systematic refinements to balance diagnostic performance and computational efficiency. Fourth, due to the small sample size relative to the complexity of the task, we performed manual image cropping to minimize noise and improve model performance. While this helped, it introduced additional workload for human users.We anticipate that future models will be able to focus on the key region of interest through iterative training as more data become available, enabling a fully automatic pipeline and enhancing user convenience. Fifth, we did not include a control group with healthy anterior ocular segments, as the model was specifically developed to assist in differentiating ocular surface tumors when a lesion is present. Nonetheless, future iterations will aim to incorporate a wider spectrum of conditions to improve diagnostic versatility and generalizability.Lastly, while UBM is not yet widely available in all clinical settings, its unique imaging capabilities offer valuable diagnostic insights, especially in ambiguous cases. Our model demonstrates that AI applied to UBM can outperform human experts in differentiating ocular surface tumors. Nonetheless, future studies should explore multimodal approaches by incorporating clinical photographs and other imaging modalities to improve diagnostic accuracy and broaden applicability.

To address these limitations, future studies should be conducted to enhance model performance and improve generalizability by refining our AI model, incorporating additional clinical data sources, and expanding training datasets. The next step involves deploying and validating the model in clinical settings, potentially integrating an active learning framework that continuously improves based on clinician feedback. The ultimate goal is to establish a reliable, efficient, and transparent AI-assisted system for ocular tumor evaluation and management.

In conclusion, this study demonstrates the feasibility of applying AI to assist ophthalmologists in the differential diagnosis of ocular surface tumors using UBM scans.The deep learning model outperformed human specialists in distinguishing between benign and malignant tumors, offering a promising diagnostic adjunct for clinical practice. The implementation of heatmaps enhances model transparency, providing interpretability crucial for clinical acceptance. Moreover, the model’s diagnostic performance was consistent across patient subgroups, supporting its fairness and potential to reduce diagnostic disparities. Future research will focus on refining the model with larger, more diverse datasets and integrating AI into routine ophthalmic practice to optimize patient care.

## Abbreviations

UBM: Ultrasound biomicroscopy
AI: Artificial intelligence
OSSN: Ocular surface squamous neoplasia
CIN: Conjunctival intraepithelial neoplasia
SCC: Squamous cell carcinoma
CNNs: Convolutional neural networks
HR-OCT: High-resolution anterior segment optical coherence tomography
AUROC: Receiver operating characteristic curve
